# A Narrative Review of the Role of Economic Crisis on Health and Healthcare Infrastructure in Three Disparate National Environments

**DOI:** 10.3390/ijerph17041252

**Published:** 2020-02-15

**Authors:** David M. Claborn

**Affiliations:** Master of Public Health Program, McQueary College of Health and Human Services, Missouri State University, Springfield, MO 65897, USA; davidclaborn@missouristate.edu; Tel.: +1-417-836-8945

**Keywords:** economic collapse, workforce, tuberculosis, disaster

## Abstract

The collapse of a country’s economy can have significant impacts on the health and healthcare infrastructure of the country. This paper compares the collapse of three national economies from widely separated regions: Venezuela, Zimbabwe, and the countries of the former Soviet Union. Despite significant differences in the environments and cultures of these countries, there are some common variables and outcomes shared by most of the countries including effects on healthcare workforce, disproportionate effects on marginalized populations, and resurgence of certain infectious diseases.

## 1. Introduction

It is a truism to state that an economic collapse at the national, regional, or international level can directly affect the public’s health. However, the effects in different countries are rarely uniform and often the opposite of what observers might expect. As an example of an unexpected outcome, mortality rates in the United States declined during the Great Depression [1]. Observers have proposed several non-economic reasons for this decline, including improved health education and reduced alcohol use during the concurrent Prohibition, but it is still true that reduced mortality during an economic collapse is not an expected outcome. Another example of an unexpected outcome is the decline in the prevalence of human immunodeficiency virus (HIV) infections between 1997 and 2012 during the severe economic collapse in Zimbabwe [2]. Despite these examples, collapses of economies are often associated with declining levels of public health often exemplified by epidemics of re-emerging diseases and the collapse of the healthcare infrastructures, including the workforce of healthcare professionals. 

There are numerous examples of such consequences, many of them associated with conflict and its effects on the local and national economies. An example of the deleterious effects on the public health workforce is provided by the twenty-year insurgency from 1986 to 2006 in parts of Uganda. During that time, the majority of healthcare workers in affected parts of Uganda fled the country. Those who remained were often traumatized to the point of having great difficulty in performing their jobs [3]. Similarly, an 11-year civil war in Sierra Leone in which 50,000 people were killed and two million were displaced resulted in the near-total destruction of the healthcare infrastructure [3]. An outbreak of Ebola emerged from this environment, making the working environment for health workers even more difficult. Perhaps one of the most striking examples of a healthcare infrastructure destroyed by conflict and economic distress is provided by the Khmer Rouge regime in Cambodia. Between 1975 and 1978, the Khmer Rouge destroyed the economic infrastructure of Cambodia in an attempt to make the country into an agrarian society. Certain sections of the population were targeted for “resettlement and re-education”. This targeted population included many of the intellectuals and healthcare professionals and these populations were decimated. About two million people in this small country died of executions, disease, and starvation [3]. Although conflict can deleteriously affect nearly every aspect of the public’s health, economic collapse can occur in the absence of conflict and can also have disastrous results [4]. This paper will review and analyze relatively recent events in the former Soviet Union, Venezuela, and Zimbabwe as examples of how collapse of an economy can deleteriously affect a population’s ability to maintain its health and health system, even in times of relative peace. These three examples provide opportunities to compare very different environments involving the impact of economic collapse on health infrastructure and the general health of populations. The purpose of this review will be to identify common variables that may be expected in other situations involving economic collapse, with or without conflict, and the effects these conditions have on the public’s health. These countries reflect very different histories and environments, some being in tropical regions and others in temperate. Cultural, governmental, and ethnic backgrounds between the three countries also vary greatly, so it is challenging to draw universal conclusions about how economic collapse affects a specific country’s public health from these examples. For instance, the resurgence of neglected tropical diseases in Venezuela is very different from the health issues experienced by the countries of the former Soviet Union. However, there are some common factors such as impacts on healthcare workforces and the resurgence of certain communicable diseases. This paper will compare the effects of economic collapse on the public health in Venezuela, Zimbabwe, and the countries of the former Soviet Union.

## 2. Materials and Methods 

This narrative review of the literature used two primary sources for access to the literature regarding the effects of economic collapse on public health: PubMed Central^®^ and Academic Search Complete, an EBSCO host product housed in the Missouri State University Library system. In each system, the following words or word groups were used to search for appropriate literature: “economic collapse” and “public health”. These words were used in combination with the names of the three countries of interest: Zimbabwe, Venezuela, and the Soviet Union (or USSR). These countries were chosen because they exemplified very large economic crises but were each on separate continents and were very different in culture and ethnicity. Selected publications were limited to those from the last ten years from the date of the search, though a couple of older publications were used to provide some historical background on the Soviet system. 

## 3. Results

### 3.1. Countries of the Former Soviet Union

The economic collapse of the Soviet Union (USSR) probably started in the late 1980s, but the breakup of the USSR government in 1991 worsened the situation for the emerging countries left after the dissolution [5]. In Russia, the economy had recovered substantially by 2009. As the union dissolved, several independent countries emerged that had to build their own economies and infrastructures, including their own healthcare and public health systems. These efforts would have been challenging in any situation, but they were made even more difficult by a worsening economy for the entire region. Several Central Asian countries suffered reductions in gross domestic product between 33% and 60%; public health spending also fell sharply [6]. These reductions were due in large part to the removal of Soviet support after the final dissolution of the USSR in 1991. 

Soviet Russia utilized the Semashko healthcare system that was socialist in structure, with a centralized and hierarchical structure that provided health care to all citizens. It was particularly successful in addressing infectious disease, but by the 1980s the quality of care was considered low, with shortages of medical equipment, drugs, and supplies for diagnostics. Training of medical personnel was considered mediocre and pay was poor. Soviet physicians earned only 70% of the income of an average non-farm worker in that country [7]. Even before the dissolution of the Soviet Union, the citizens had poor life expectancy and high early mortality rates. The government paid nearly 92% of all medical expenditures, yet key indicators in 1990 indicated severe problems with the system. For instance, maternal mortality in the Soviet Union was nearly 7 times that of the U.S. Male life expectancy was nearly seven years shorter [7]. With the economic collapse and subsequent dissolution of the USSR, the healthcare situation became worse for many of the emerging republics. The economic collapse was accompanied by the collapse of the Soviet Union, so it is difficult to determine proximal causes for many of the public ills that occurred in the emerging countries during the 1990s. For instance, the rapid social changes and open borders contributed to a significant increase in organized crime, with subsequent increases in health problems associated with illicit drug use and poor-quality alcohol [8]. Nevertheless, several other events were obviously linked to the deteriorating economy. The collapse of the centrally planned healthcare program quickly led to the development of a black-market economy for healthcare services. Tips and gifts made up to 30% of the total salary for healthcare personnel. At the same time that there was a rising popular demand for health services, the governments’ response in some cases was to increase regulations to reduce access to the services. In part, this response was due to decaying healthcare infrastructure. Resources were inadequate and most hospitals had a severe shortage of equipment. This was particularly true in rural areas, which experienced an exodus of doctors into urban centers. Most of the new countries suffered severe shortages of pharmaceuticals as they were suddenly subject to Western market prices. The eastern European country of Poland was spared some of this shortage because it had a developed national pharmaceutical industry; however, it was unable to provide enough of the products to prevent shortages in the countries of the former Soviet Union.

A detailed analysis of one rural area in Russia provides some idea of the impact both the economic collapse and the political dissolution caused in such communities. Chukotka is a region of the Russian Federation in the north-eastern side of the country; about half of it lies within the Arctic Circle [9]. During the Soviet era, the local economy was stable and depended on local deposits of oil, gas, gold, platinum, silver, and other minerals. Salaries were higher than in other arctic regions of the USSR and higher than the national average, so there was a sufficient supply of professionals, including healthcare personnel. Many immigrants moved into the area to take advantage of the job market and high pay scale. This all changed with the collapse of the Soviet Union in 1991 when Chukotka suffered an extended period of depopulation and economic decline. Much of this was due to the closure of military facilities and many of the local mines. From 1993 to 2013, the population of Chukotka decreased by over 65% due in large part to the departure of non-indigenous people, including military members and their families. Disease rates of tuberculosis and sexually transmitted diseases increased rapidly as the demographic situation changed. Many of these changes in disease rates were attributed to the departure of low-risk groups and the continued presence of groups with historically high rates of disease, specifically some of the indigenous groups [9]. The indigenous groups had a long history of suffering from poverty, alcoholism, suicide, and higher rates of infectious disease. Crude mortality rates in the indigenous populations of the region had steadily decreased between 1976 and 1991, but starting in 1992, they steadily increased and remained above 1987 rates through at least 2009. Unexpectedly, high rates of chronic otitis, chronic bronchitis, and pneumonia were observed in the local population, along with gonorrhea, which occurs at a rate up to six times that of the rest of Russia. The indigenous population was also at increased risk of parasitic disease associated with consumption of fish. Surprisingly, some indigenous populations reported a continued reduction in the rate of tuberculosis, though some observers suggested that this was due to inadequate detection efforts; others thought the reductions might be real and be due to reduced contact with outsiders. 

In short, Chukotka suffered an economic collapse after the breakup of the Soviet Union that resulted in large demographic changes and resultant degradation of the public health situation. Due to improvements in governance [9], the economy has rebounded in recent years with a resultant increase in the population. However, there are residual problems with the health of indigenous populations that are associated with unemployment, poverty, suicide, alcoholism, and certain infectious diseases such as tuberculosis and sexually transmitted diseases. 

Results across the various former republics varied, but in Central Asia, the transition for the centralized Soviet system to smaller national systems was very difficult. The Central Asian Post Soviet (CAPS) countries experienced a reduction in gross domestic product of between 33% and 60% [6]. Public health spending fell sharply to as low as 2.1%. The reasons for these decreases are complex but undoubtedly were due in large part to the removal of Soviet support. The central Asian countries, including Kyrgyzstan, Tajikistan, Turkmenistan, and Kazakhstan all had limited histories with donors, who in turn had difficulty working in the national settings with limited transparency. The Soviet system had developed with large infrastructure costs that became difficult to maintain after the collapse. Moreover, the Soviet emphasis on curative medicine required continuous support and was not sustainable.

Prior to the collapse, the Soviet healthcare system had been known for inefficiency and neglect; however, it was able to achieve a steady decline in the rates of tuberculosis. There were specialist tuberculosis hospitals and dispensaries across the USSR that were effective at diagnosing and treating the disease. After the demise of the USSR, some countries were able to make a transition to health insurance and general practice with increased out-of-pocket payment [5]. However, in many places, there was a decline in social conditions and living standards accompanied by unemployment and financial insecurity. These contributed to proximal causes of increased TB rates, including alcoholism, crowded housing, malnutrition, tobacco smoking, and increased incarceration rates. The incarceration was strongly associated with multi-drug resistant tuberculosis (MDR-TB) rates that have been the source of much public health concern [10]. Observers have also expressed concern about epidemics of HIV, hepatitis C, and syphilis in incarcerated populations; in Kyrgyzstan, the HIV-infected population was reportedly concentrated 34-fold higher in prisoners [11]. 

In general, the reconstruction of national health systems during the economic collapse concurrent with the demise of the Soviet Union provided unique challenges to the emerging governments. These challenges varied by country, but those countries that maintained sufficient disease surveillance and monitoring programs were more likely to be able to mitigate the public health effects of the economic collapse. Of course, this observation could be explained by the possibility that those countries that were better situated to maintain surveillance were wealthier and so better able to weather the economic situation. Nevertheless, some of the emerging nations ignored the importance of drug-resistant tuberculosis and other emerging diseases, and this resulted in a significant public health crisis in much of the former Soviet Union, most notably in the prison systems.

### 3.2. Zimbabwe

After independence in 1980, Zimbabwe’s economy eventually went into a long decline, but a precipitous economic collapse occurred between 2000 and 2009. By 2008, the country suffered hyperinflation of up to 500 billion percent which destroyed the local currency [2]. The gross domestic product of the country decreased by 40%, leading to significant reductions in living standards and the availability of public health services, including clinical health care. 

Several government policies contributed to the severity of the economic collapse including land re-distribution practices, unstable currency policy, and government price controls [12]. According to some observers, government policy was particularly culpable for an epidemic of cholera in 2008–2009 [13]. That epidemic resulted in more than 98,000 cases and more than 4000 deaths. The government of Zimbabwe was accused of several human rights abuses that exacerbated the severity of the disease including refusal to fund municipal budgets in regions that did not support the ruling party, and the politicization of water. As a result of these policies, water treatment was unfunded in some large cities, including the capital of Harare. Sewage treatment was also unfunded and much of the untreated sewage went into the main reservoirs. It is an accepted rule in public health that maintaining a proper infrastructure for sewage and water treatment is essential to the public health of a community, especially for combating the fecal–oral route of disease transmission. An outbreak of cholera in this situation could not have been surprising. Local municipal water authorities were eventually subsumed within a national system, then used to punish regions that supported the political opposition. The ruling party politicized water and food. Water rates were increased exorbitantly in regions that did not support the party and revenue was diverted to military and security forces. In addition, requisite chemicals for water treatment became unavailable. The poor state of the healthcare system during these crisis years contributed to the epidemic by failure to properly treat the disease, leading to a high case fatality rate up to five times that of the international standard [13]. There was a near-total collapse of the health care delivery system, with most wards in large public hospitals being closed. Some of the best hospitals closed due to a lack of water supply as well as the absence of essential medicines and supplies and staff absenteeism. By many accounts, the ruling party’s focus on power rather than governance was the fundamental cause of the economic collapse, but its actions during the crisis years made the effects even worse [13]. 

The economic collapse led to a variety of changes and adaptations by the populace to provide income; the sex trade was one industry that was greatly affected. At least some of these changes led to greater risk to women involved in the trade, and it probably led to a greater number of women involved as well. Female sex workers had always been disproportionately affected by the HIV/AIDS epidemic and this continued during the crisis years in Zimbabwe. What did change was an increase in “transactional sex” as opposed to “commercial sex” [2]. The difference in the two categories as defined in the referenced literature is that women involved in transactional sex do not see themselves as sex workers, but instead may use sex to obtain necessary commodities such as food or soap. It is usually seen as poverty-driven. Observers noted that the HIV epidemic, coupled with the economic collapse, led to changes in the location of where prices for sex were negotiated. Bars had previously been the sites of most negotiation, but with reduced access to money necessary to purchase alcohol, the sites changed to places where commodities used in the transactions were available. Much of the trade became associated with the trucking industry, reportedly with the delivery of food. Some of those involved described the change from professionals to amateurs. 

As with other countries undergoing economic collapse, Zimbabwe experienced a resurgence in TB. Since the onset of the HIV/AIDS pandemic, it has become very difficult to separate these two phenomena, especially in times of crisis, but TB incidence increased by 35% in 2008 when compared to a baseline from 2003–2007 [12]. In Zimbabwe, the majority of TB cases in some regions were co-infected with HIV, suggesting that any modern study of crises must consider the impact of the HIV pandemic on a variety of variables. However, in sub-Saharan Africa, there has been a documented relationship between poverty, overcrowded housing, and higher TB prevalence [12]. During the Zimbabwean economic crisis, malnutrition as a result of poverty also played a role. Accurate estimates of TB rates, however, were nearly impossible to acquire during the crisis as healthcare facilities closed and disease reporting ceased in many areas. In addition, the diagnosis was based on clinical, smear positivity, and radiological data rather than the definitive culture confirmation. Researchers noted that food insecurity with malnutrition as a result of economic collapse can significantly increase TB incidence, and this conclusion is especially true in the context of an ongoing epidemic of HIV/AIDS.

The economic collapse also affected the availability of healthcare as a result of deleterious changes to the healthcare workforce [14]. An assessment of staffing levels in 2009 showed that only 34% of physician positions were staffed, resulting in a physician to 1000 population ratio of 0.067. The World Health Organization’s recommendation at the time was 0.2. All categories of health workers reported were understaffed, with the lowest numbers seen in physicians, environmental health officers, pharmacists, and certified laboratory technicians. The reductions in populations of health professions were attributed to “out migration”, transfer to private sector or non-health related fields, early retirement, HIV/AIDS, and death. The replacement rate was insufficient due to impacts on the education and training of health professionals. As observed in other economic crises, rural areas suffered disproportionately with regard to the availability of professional healthcare workers. The greatest deficit in rural and urban caregivers was in those who provided care to pregnant women, new mothers, and children. In fact, government policy at the time restricted the practice of midwives and this policy may have exacerbated the shortage [14]. The lack of proper care for children was particularly obvious due to statistics on under-five mortality. In 2000, the Millenium Development Goals (MDG) established a target of reducing under-five mortality by two-thirds. Globally, this measure of public health was reduced by 51% by 2015, but in some countries of sub-Saharan Africa, including Zimbabwe, reductions were much less. Between 1990 and 2015, under-five mortality in Zimbabwe dropped from 75.8 to 70.7 per 1000, a small decrease compared to global trends. 

### 3.3. Venezuela

In November of 2019, Reporter C.K. Carugo wrote the article “My Socialist Hell: Living in Post-Electricity Venezuela” and stated that 20-years of the “Bolivarian Revolution” had resulted in long-term underinvestment in the electrical power infrastructure in Venezuela, leading to blackouts for the entire country [15]. Lack of power and poor management of the water system also led to the lack of running water during parts of each day for over three years in Caracas, the nation’s capital. The article documented a long-term economic downturn in the formerly prosperous and wealthy South American country. The economic situation has had significant impacts on the public health of Venezuelans. Another contemporaneous news article by Anatoly Herrera [16] stated that the collapse of the water system in Venezuela had led to an infant death rate due to diarrhea nearly six times that of a period 15-years earlier Perhaps because of this and other extraordinary increases, the government quit releasing public health data, which had historically been unreliable anyway. The situation outside Caracas was reportedly worse, with hepatitis A rates at times 150 times the normal rate. 

These two news reports are not peer-reviewed nor confirmed by government sources and at least one was posted on a highly partisan site. However, the PRO-MED website, an internet-based reporting system disseminating information about outbreaks of emerging infectious diseases, also reported a national epidemic of malaria as well as outbreaks of diphtheria, trypanosomiasis, pertussis, nosocomial chickenpox, cholera, and rabies. Reports of such diseases are not unexpected given that Venezuela is a tropical country, but there is little doubt that the current economic situation in that country has contributed to a public health disaster. That disaster has been called a “humanitarian crisis of post-war dimensions” [17].

Political perspectives influence descriptions of what caused the economic collapse. The populist policies of Hugo Chavez and his successor, Nicolas Maduro, have been blamed by many for the economic situation. The economic collapse had become obvious by 2014 and has continued into the present. Specific causes may include an over-reliance on petroleum during a world-wide reduction in oil prices, and reckless public financing followed by irresponsible printing of money that led to massive public debt, overvalued currency, and inflation. However, some variables existed before the populist revolution led by Hugo. Corruption has been an issue in Venezuela for many years. Lack of access to healthcare, education, and housing was also important. As the situation worsened, external investors and donors withdrew their money and the collapse occurred throughout the nation [17]. An important health issue during this period of economic distress in Venezuela has been the resurgence of vector-borne and neglected tropical diseases. This has happened in the environment of a regional epidemic of arboviral diseases like dengue, chikungunya, and Zika virus disease. However, the resurgence of vector-borne diseases in Venezuela has greatly exceeded what has happened in neighboring countries [18]. For instance, malaria rates in the rest of South America have decreased by 50% in recent years, but there has been a 300% increase in malaria cases in Venezuela. The situation is complex and it would be inaccurate to state that the economic situation is the only cause of the increase in malaria rates. Much of the increase in malaria rates has been attributed to larger numbers of migrants, many associated with illegal gold mining, who have moved into inadequate housing in the mining regions. The author observed gold mining in a remote jungle location in Venezuela many years ago and noted that work continued 24 h a day, so workers could not retire to a screened or insecticide-treated house during the peak periods of mosquito activity. Housing often consisted of a tin-roofed shack with no walls, and workers slept in hammocks with no mosquito nets. The housing situation for these miners was indeed inadequate (Claborn, personal observation). Not surprisingly, the epidemic has resulted in exporting cases to Brazil, Guyana, and Columbia. Malaria, however, is not the only emerging vector-borne disease in Venezuela.

American trypanosomiasis (or Chagas disease) is a parasitic disease spread from zoonotic reservoirs to humans by the cone-nosed bugs (family *Reduviidae*). It is a disease that thrives in tropical environments where the mammalian reservoirs live. Those reservoirs include rodents, opossums, bats, and other mammals. In Venezuela, Chagas disease has emerged in an urban form in the Caracas valley, which includes the nation’s capital, Caracas [19]. The population of the valley has increased significantly in recent years due to migration from other parts of Venezuela, leading to large sections of the city having poor hygiene standards and low socioeconomic conditions. The internal migration to Caracas has occurred due to conditions that are better than they are in other parts of the country, but urban slums house much of the population near polluted rivers that cross the valley and the city. The valley had supported mammalian reservoirs and vectors long before the human population became as large as it is today, and the progressive modification of the environment allowed these mammals, especially the opossum, to adapt to the urban environment. Public health workers have called for improved socio-sanitary programs including vector control, housing initiatives, and medical treatment to address this developing disease situation, but such programs are not likely to improve significantly in the current economic environment. 

Poverty is often described as a social determinant of vector-borne disease in the tropics and it continues to be important in Venezuela; however, political and economic instability have become major influencing variables as well. Inadequate funding and direct actions on the part of government have had several effects that influence the rate of vector-borne diseases [17]. Lack of funding and disinterest in reporting actual disease rates have reduced epidemiologic surveillance and disease reporting, preventing the appropriate targeting of disease control initiatives. Similarly, many government workers have gone long periods of time without pay. Human migrations associated with illegal mining have displaced populations and increased their exposure to disease vectors. A shortage of fuel for vector-control initiatives has reduced the degree to which these actions occur. Finally, a shortage of effective insecticides and anti-parasitic prescriptions has reduced the protection the Venezuelan populace has from these diseases. 

Another aspect of public health in Venezuela that has degraded is the national ability to deal with cancer. Patients with complex health conditions like cancer often cannot obtain adequate treatment. In Venezuela, hospitals are reportedly bare of supplies, equipment, and pharmaceuticals [17]. This is not necessarily due solely to the unavailability or expense of the drugs, but also to rampant crime that results in the theft of the necessary supplies for eventual sale on the black market. Inflation was up to 700% annually at one time, so such purchases bankrupt families. The pharmaceuticals most in demand are antibiotics, anti-hypertensives, contraceptives, and analgesics. Cancer cases are particularly vulnerable not only due to lack of needed drugs but also due to incompetent or inadequate care. In some cases, the government mandated specific treatment regimens that were contra-indicated. 

The situation with cancer treatment reflected that of general clinical care in Venezuela. As the economic situation degraded, government agencies started falsifying health statistics and indicators. In one case, a report was so erroneous that the United Nations gave Venezuela a prize for a successful feeding initiative when in fact a famine was occurring [17]. 

The clinical health care system in Venezuela consists of three parts: the public system that is open to all, the national insurance system, and the private health system. When Chavez became president, his government instituted populist measures to address significant inequities in healthcare access in the country, but the entire system suffered greatly as a result of rampant corruption and mandated budget cuts during these changes. In April 2019, Human Rights Watch issued a report about the humanitarian crisis that summarized the situation with some startling statistics [20].

1. There has been an exodus of about 3.4 million Venezuelans.
2. Between 2008 and 2015, there was only one case of measles reported in Venezuela; since 2017, there have been more than 9300 cases.
3. Between 2006 and 2015, no cases of diphtheria were reported in Venezuela. Since 2016, about 2500 suspected cases have been reported.
4. In 2014, about 6000 cases of tuberculosis were reported; in 2017, 13,000 cases were reported. This is the highest rate of tuberculosis in Venezuela in 40 years.
5. Venezuela is the only country in the world in which HIV+ persons have had to discontinue treatment with antivirals due to the unavailability of the drugs. About 87% of these patients registered with the state are not receiving appropriate treatment.
6. Venezuela is the only country in South America in which maternal and infant mortality rates have returned to pre-1990 levels.

This same report placed the blame for the worsening situation on the Maduro government primarily due to its denial of the existence of a crisis and to its refusal to allow international aid to alleviate the public health situation and the concurrent food security crisis. Other aspects of the political situation in Venezuela seem to perpetuate the economic collapse, especially the competing perspectives of the National Assembly and the National Constituent Assembly, as well as competing claims to the presidency by Nicolas Maduro and Juan Guaido. Whatever the proximal cause of the economic crisis, it is obvious that the crisis continues. 

## 4. Discussion

The countries of the former Soviet Union, Zimbabwe, and Venezuela are very different nations, each on a different continent and supporting very different populations with a variety of ethnicities, cultures, and economies. The fact that economic collapses occurred across this spectrum of countries seems to be about the only thing they have in common, particularly given the fact that Zimbabwe and Venezuela are tropical countries; whereas, the Soviet Union included temperate and even large circumpolar regions. Despite these great differences, there are some factors relating to health and economic collapse that are shared by these countries. Those factors may not be universal or unique to economic collapse, but they warrant further discussion.

First, economic collapse appears to have strong and long-lasting effects on the health workforce and the health infrastructure of the countries affected. In Venezuela, the effects were obvious not only in the absence of pharmaceuticals and supplies needed for clinical care but even to the availability of insecticides required for control of vector-borne disease. Even the healthcare workers associated with international charities may be affected. When the charities moved to other countries to address other developing needs, many workers from the collapsed economies followed the charities to obtain better-paying jobs [3]. The availability of well-trained professionals during an economic collapse is severely limited, and this extends across the spectrum of professionals, from physicians to environmental health specialists.

Second, national health systems have difficulty competing for the time of professionals with dual responsibilities in the public and private sectors because the professionals tend to prioritize their private patients and responsibilities. This was best observed in Zimbabwe [14] but is a common theme in all three countries discussed here. 

Third, the centralized national health systems may be most vulnerable to system-wide collapse. They are subject to direct government supervision and mandates that can result in inefficiencies and inappropriate care, as was described for cancer treatment in Venezuela [17] but more importantly, all sections of the centralized system are dependent on the same government and economic system. When one collapses, the others are also in danger of collapse. In most of the countries described in this paper, the national healthcare system co-existed with a private healthcare system. When the national health systems collapsed, the population turned to the private system that continued to function to some extent, though probably with severely reduced capabilities. In fact, some of the resources from the national health system were often appropriated for use in the private system. Even with these misappropriations, many patients had to turn to traditional healers, alternative and herbal medicines, and religion. Traditional medicine was a common factor in all three countries described here.

Fourth, rural areas tend to suffer greater problems with finding healthcare. Limited medical resources may be pulled out of rural areas to maximize the benefits of the resource. Physicians and other healthcare professionals in rural areas may have to move to urban areas for financial reasons, or they may be moved by their health system to address increased needs in urban centers. This was a theme across all countries described in this paper. In addition, certain diseases like malaria tend to present greater risk in rural environments, so the disease risks may increase for rural populations. This may not be the case in the urban areas of Venezuela which suffer from the collapse of municipal services like water, sewage treatment, and electrical power. The “politicization” of water in Zimbabwe is similar. Such situations have tremendous potential for numerous and large-scale epidemics.

Fifth, although the specific disease risks vary greatly across various countries, from tropical to circumpolar, one disease seems to present a common threat to many: tuberculosis. From the northern climes of Russia to sub-Saharan Zimbabwe, tuberculosis resurged during the periods of economic crisis. For the medical profession, this must be a great disappointment given the great successes achieved in controlling this disease in the latter part of the 20th century. There are at least two factors that have contributed to the resurgence of tuberculosis, including the concurrent HIV/AIDS epidemic that degrades the immune systems of many victims, and the development of multi-drug resistant strains of the tuberculosis bacterium. In times of crisis, when appropriate treatment is difficult to obtain, and malnourished people are crowded together into inadequate housing, tuberculosis re-emerges. It is also a very serious threat to the incarcerated. 

Sixth, marginalized groups will suffer the health impacts of the economic collapse disproportionately. In Chukotka, it was the indigenous populations who remained in the regions following the economic collapse and the dissolution of the Soviet Union, and they suffered disproportionately from poverty, unemployment, alcoholism, tuberculosis, and sexually transmitted infections. In Zimbabwe, commercial sex workers and those involved in “transactional” sex were at greater risk of poverty and HIV infection. In Venezuela, the migrants involved in gold mining or who abandoned the countryside for the crowded, unhealthful urban slums of Caracas were at higher risk for a variety of diseases. Many observers have noted that displaced populations are at a particularly high risk of disease [21]. Indigenous peoples are often marginalized and vulnerable to fluctuations in disease risk seen during the economic collapse.

## 5. Conclusions

Public health authorities during times of economic crisis must prioritize their personnel, time, resources, and finances in such a way to maximize the benefits to the supported population. The needs are not the same for all situations, so decisions must be appropriate. Nevertheless, there are some recommendations that come out of the comparisons presented here. Foremost of these recommendations is the need to maintain effective disease surveillance and monitoring, especially in regions and populations that are most at risk of diseases with a history of rapid emergence [5]. Authorities in some of the countries described here ignored the emergence of drug-resistant tuberculosis until it was nearly too late. Appropriate surveillance and monitoring could have provided the information needed to mitigate the disease outbreak. In Venezuela, the need for better disease surveillance is particularly obvious. Some observers have commented on the resurgence of measles and diphtheria in Venezuela during the current economic crisis there. Indigenous people in the Amazon region are most at risk. Perhaps more alarming is the potential for re-emergence of poliomyelitis that has been absent from the Americas for 20 years. There is a current need to improve surveillance of vaccine-preventable diseases that will drive the purchase and maintenance of appropriate vaccine stocks [22].

A decline in good health is a result of several variables such as one’s exposure to risk factors or one’s behavior. However, it is also true that health depends on social cohesion (informal welfare) and social protection (formal welfare) [1]. These are elements of societies that can be used to provide support to populations, even though reduced government finances may limit the availability of social protection. Government spending has been shown to suppress suicides, though it must be noted that excessive spending is consistently reported as one of the factors that lead to economic collapse. Employment assistance may, however, be a government service that is important and productive in mitigating the public health effects of an economic crisis. Unemployment is linked to increases in daytime drinking and perhaps tobacco smoking. Social cohesion in the form of sports clubs, trade unions, religious organizations, and families maybe some of the most effective supports for populations during an economic collapse.

Most of the countries described here have progressed through a degree of recovery. For many of the countries of the former Soviet Union, this required a transition from a centralized economy with a centralized healthcare system to a variety of systems, some involving insurance schemes and others national health systems or both. Observers suggest that such transitions require an investment in health systems even while realizing the reduced availability of capital. They also recommend welfare-to-work programs and a renewed focus on education. The economic crises and the responses that each of these countries went through are not necessarily unique to those counties, so the common themes discussed here may have applications to other countries experiencing economic crises.
